# 

**Published:** 1890-12-20

**Authors:** 


### NOTICE TO CORRESPONDENTS.

All MS., Letters, Books for Review, and other matters intended for the Editor should be addressed The Editor, The Lodge, Porchester Square, London, W.

The Editor cannot undertake to return rejected M.S., even when accompanied by stamped directed envelope.

**Notice to Advertisers and Subscribers.**

All Advertisements, Orders for Copies of The Hospital, and Business Communications should be addressed to The Publisher, *not to the Editor*, The Hospital, 140, Strand, W.C.

**Bound Volumes of "The Hospital."**

Vol. VI., containing full page portraits of T.R.H. the Prince and Princess of Wales, and Miss Florence Nightingale, is still obtainable.

Vol. VII. and all the earlier volumes are also for sale.

Orders will be received for Vol. VIII., 4s. post free. Cases for binding may be had of the Publisher, at 1s. 6d. each.

**To Residents, Secretaries, and Matrons.**

The Editor will be glad to have the Regulations and each Report as issued by Asylums, Hospitals, Convalescent and Nursing Institutions, as well as those of other Charities, and an early copy in duplicate of all Appeals and Festival Notices, etc., addressed to *The Editor, The Lodge, Porchester Square, London, W.* Any superintendent, secretary, matron, or other chief official who regularly sends such information may be registered, on application to the Publisher, to receive free of cost a cover for binding The Hospital in half-yearly volumes.

---

**BURDETT'S HOSPITAL ANNUAL FOR 1890**

May be obtained of the Publisher, The Hospital Offices, 140, Strand, W.C., price 2s. 6d. limp boards, or 3s. 6d. cloth. All orders must be accompanied by a remittance.

### Scraps and Gleanings.

The Prince of Wales has sent a present of game to St. George's Hospital.

Dr. Behring states that he has found no cure for diphtheria in human beings as yet.

A sale of work has been held in aid of the Weston-super-Mare Hospital. £160 was taken the first day.

Lord Tredegar opened the Newport Infirmary bazaar on December 11th; the bazaar was very successful.

Mr. Montagu Williams last week gave judgment against 42 houses in Bethnal Green, said houses to be closed.

Alderman Charlesworth has been elected chairman of the Hanley, Stoke, and Fenton Joint Hospital Board.

The temporary Woman's Hospital at Southampton has done such service that it is proposed to start a permanent institution.

Dr. Image has published a letter from one "Sarah Drew," who complains of the food served to the nurses at the Suffolk General Hospital.

Queen Charlotte's Lying-in Hospital has recently been awarded £100 by the Grocers' Company and fifty guineas by the Mercers' Company.

The Duchess of Teck lately helped with a bazaar at Richmond in aid of the Society for the Prevention of Cruelty to Animals. Nearly £400 was taken.

Subscriptions to the amount of £400 were announced at the festival of the London and South-Western Railway Widows and Orphans' Benefit Society.

Colonel Seely has offered a conditional £10,000 towards supplying Nottingham with a proper convalescent institution. This is wise and munificent generosity.

Throughout the diocese of Hereford Hospital Sunday was held on the First Sunday in Advent. The Mayor and Corporation attended Hereford Cathedral in state.

Professor Tessier, of Lyons, has been to Russia to study influenza; he says it is a growth of the Russian soil, and the microbe is found in the mud of the village streets.

The Right Reverend Dr. Barry preached at St. Thomas's Hospital on December 11th in aid of the Medical Missionaries of St. Thomas's, four of whom are working in the mission field.

Mrs. Mills, the wife of the Hospitaller of St. Thomas's, has carried on a working party ever since 1883. It has contributed 1,415 garments to the hospital. Meetings are held on alternate Fridays from November to May.

Dr. Tessier is charmed to see how Russian women who have studied medicine in Switzerland and France are bringing up the standard of medical practice in rural Russia and dispelling the awful ignorance of the people.

Tom Smith will give this coming Christmas 20,000 prettily designed crackers for distribution among the poor of the London workhouses and hospitals. Last year the same firm contributed 10,000 to the "Truth Toy Fund."

Dr. Aldridge has been elected assistant physician, and Mr. Rellow dentist to the Royal South Hants Infirmary. Major Crabbe has been elected trustee in place of Dr. Lake, resigned on account of the new nursing scheme.

The members of the Leprosy Commission have arrived at Bombay, and have been welcomed with marked demonstrations of approval and good feeling. The Commission consists of Dr. G. A. Buckmaster, Mr. A. Kanthack, and Dr. Beaven Rake, of the Leper Asylum, Trinidad.

There is an outcry in the Paris press against the Board of Health for recommending the Government to authorise the use of Dr. Koch's lymph. Deputy Arène, in the *Paris*, recommends the doctors to curb their curiosity and wait until it is well ascertained at Berlin that the lymph is not a virus.

The Leprosy Commission has examined many cases at Hyderabad, and it is stated that a few of them were declared to be unequivocal examples of leprosy. It is understood that the Commission, after spending six months inspecting cases in the plains, will pass six months at Simla, carrying out experiments in connection with the cultivation of the leprosy bacillus.

The following resolution was passed by the great meeting at the Mansion House to consider the treatment of the Jews in Russia: "That, in the opinion of this meeting, the renewed sufferings of the Jews in Russia, from the operation of severe and exceptional edicts and disabilities, are deeply to be deplored, and that in this last decade of the nineteenth century religious liberty is a principle which should be recognised by every Christian community as among the natural human rights."

At a crowded meeting of Trades Unionists last week, Mr. Tom Mann moved a resolution that in view of the large number of unemployed workers in the metropolis, the London County Council "might forthwith adopt some scientific and sanitary method of utilising the sewage of the metropolis instead of the present system of waste and pollution of the River Thames, whereby a large amount of labour could be profitably employed to the advantage of the ratepayers generally; and that a deputation from the council wait upon the County Council to urge the adoption of the foregoing proposal."

At the annual meeting of the Southampton Hospital Sunday Fund the Rev. J. M. G. Owen brought forward the following motion: "That every charity desiring to participate in future apportionments of this fund should be required to admit on its management direct representatives chosen and appointed at the annual meeting of this fund, such representatives to be chosen on the basis of the last previous apportionment as follows: To every charity one delegate. Where the sum voted be over £50 and under £200, two delegates; and where the sum voted be over £200, one delegate for every additional £200."

### The Student of Medicine.

[All communications for this Supplement should be addressed to The Editor of The Hospital, at 140, Strand, with the words, "Students' Column," written in the left hand top corner of the envelope.]

### EDITORIAL NOTES.

English Sports on the Continent.—Many of our sports are rapidly gaining ground in various foreign universities as favourite pastimes among the students. In France, M. Grousset recently gave an eloquent description of the game of football, which game, he says, was introduced into his country by M. Gadiot. In Germany, rowing is becoming very popular, especially at Berlin and Heidelberg. In all these sports, as on the foreign turf, the English technical terms and idioms are freely used, that is, they are spelt as in English, but pronounced as if they were French or German words. It is often very hard to recognise the words, especially so with the French accent, many of our most familiar terms being very curiously pronounced; such for instance are "offside," "outrigger," "bookmaker," "jockey," &c.

Bacteriology.—We have received a prospectus of a post-graduate course on the Pathology of Tuberculosis to be given at Queen's College, Birmingham, in January next. The lectures and demonstrations are to take place at Queen's College, and are to be delivered by Professor Gilbert Barling and Dr. G. F. Crooke. The lectures are to include the general Pathology of Tubercle with the life history of the Tubercle Bacillus and the modes of infection, of the special lesions found in the respiratory, alimentary, cerebrospinal, and genito-urinary systems, and in bone, joints, lymphatic glands, and skin. At the practical classes, those attending will be provided with an opportunity of familiarising themselves with all the details necessary for staining the bacilli in sputum, and some of the commoner tubercular lesions. We hope that before many years most of the London hospitals will give their students an opportunity of learning something of Bacteriology.

University College Botanical Laboratories.—University College has refitted the laboratory situated in the North cloister. This now serves as a museum and general elementary botanical laboratory, and accommodates forty-five students. A large room has been fitted up in the adjacent Birkbeck building as a laboratory for advanced work. This consists of a room for chemical and physiological work, a dark chamber and a small laboratory for the use of the professor. A conservatory has also been arranged with appropriate heating apparatus, and is to be used for special work on the physiological properties of plants and for the raising of seedlings.

### APPOINTMENTS.

At the Royal Westminster Ophthalmic Hospital, J. Urquhart Black, M.B., C.M. Aberdeen, has been appointed house surgeon. At the Dorset County Asylum, John Alfred Ewan, M.A., M.B., C.M. Edin., has been appointed assistant medical officer. At the Sheffield Fever Hospital, J. E. Gould, L.R.C.P. Lond., M.R.C.S., has been appointed resident medical officer. At the Public Hospital and Dispensary, Sheffield, Fred. J. Green, B.A., M.B., B.A.O., B.Ch. Dublin, has been appointed assistant house surgeon. At the Female Orphan Asylum, Biddingtown, Walter Gripper, M.B. Camb., M.R.C.S. Eng., has been appointed medical officer. At the Public Hospital and Dispensary, Sheffield, John Jeeves, L.R.C.P. Lond., M.R.C.S., has been appointed house surgeon, and Digby Cooke White, B.A., M.B., B.Ch., B.A.O., Dublin, has been appointed junior house surgeon. At the Ida Convalescent Hospital, C. Leonard Lake, M.R.C.S., L.R.C.P., has been appointed resident medical officer. At the Children's Hospital Great Ormond Street, Bloomsbury, F. T. McCann, M.B., C.M. Edin., has been appointed house surgeon. At the Royal Asylum for the Insane, Morningside, Edinburgh, James Middlemas, M.B., C.M., B.Sc., has been appointed resident pathologist. At the Leicester Infirmary and Fever House, Peter Paget, M.R.C.S., Eng., L.R.C.P., L n., has been appointed assistant house surgeon. At the Cleveland Street Asylum, William Pemberthy, M.R.C.S., L.S.A., has been appointed assistant medical officer and dispenser. At the London County Asylum, Colney Hatch, Eusebius Rouse Rouse, M.R.C.S.Eng., L.R.C.P.Lond., L.S.A., has been appointed fifth assistant medical officer. At the General Hospital for Sick Children, Pendlebury, Manchester, F. H. Westmacott, M.R.C.S., L.R.C.P., has been appointed junior resident medical officer.

### NOTES FROM THE SCHOOLS.

### EDINBURGH UNIVERSITY.

The Union debate on Wednesday, the 10th, was on the subject "That Teetotalism should be discouraged." The motion was lost by six votes. Professor Calderwood was one of the speakers.

The annual kitchen concert given by the resident physicians and surgeons of the Royal Infirmary, was held on December 11th. The concert is held in the kitchen of the Infirmary, and hence the name.

A new society has been formed—the Edinburgh University Photographic Society.

A grand organ recital was given by Dr. Greig on the afternoon of the 11th, in the University Music Class-room, and was attended by many of the students. There were also several ladies present. The first item was the chorus, trio and chorus, "God of Light" by Haydn. Some commemoration music was also played, and Dr. Greig finished his recital with the March (Alceste) by Glück. The music was very well received, and may be said to have been a complete success.

The students "go down" on December 19th.

A smoking concert was held in the Debating Hall of the Union on Friday, December 12. Sir William Muir presided during the first part of the evening, and Professors Annandale, Rutherford, Crum-Brown, and Butcher were also present. The hall and galleries were very well filled with students. The concert began by the performance of Blake's "Grand March" by the Orchestral Society (a branch of the Edinburgh University Musical Society). This was the orchestra's *début*, and was very loudly applauded. The orchestra also played "Dresdina" later on. Professor Rutherford and Dr. Meadows were encored for their songs. Professor Annandale, in the name of the members of the Union, presented a silver tea-set to Mr. Porter, the secretary of the Union during the past year, and in one of his humorous speeches proposed Mr. Porter's health, and pointed out how admirably Mr. Porter had worked. Professor Crum-Brown proposed the "Edinburgh University Union," to which Mr. W. Campbell Labon, President of the Union, replied. Before the concert, a dinner in honour of Mr. Porter was also held in the Union.

### BRISTOL MEDICAL SCHOOL.

The fund for the New Medical School buildings is growing steadily. Out of the £7,000 required £4,250 is already promised and probably in from 15 to 18 months an adequate and handsome structure will be well in progress.

At the Royal Infirmary on Saturday, the 13th inst., Dr. Frank Wethered (London), who has recently returned from Berlin and has also assisted Dr. Heron at the City of London Hospital for Diseases of the Chest, gave a demonstration of Koch's Method of the inoculation for Tuberculosis, to the medical profession of the district.

### CAMBRIDGE UNIVERSITY.

A. H. L. Newstead, B.A., of Christ's College, has been appointed to the Universities Table at Naples for six months, from December 16th.

Mr. R. T. Glazebrooke, F.R.S., who has been senior Demonstrator at the Cavendish Laboratory, for the last ten years, on resigning that appointment, is to become assistant director.

Mr. W. F. R. Weldon, M.A., F.R.S., Fellow of St. John's College, Cambridge, has been appointed by the council of University College, to the Jodrell Professorship of Comparative Anatomy and Zoology, which was held for sixteen years by Professor Ray Lankester.

### OXFORD UNIVERSITY.

A Natural Science scholarship value £60 per annum for four years will be offered for competition at Keble College, on April 18th, 1891. The examination will consist of papers and practical work in Biology and Chemistry, but a paper will be set to all candidates in Chemistry, Mechanics, and Physics, and a paper in Elementary Chemistry to those candidates who offer Biology.

### ABERDEEN UNIVERSITY.

Neither the Rugby nor Association games of football have been so popular this season. The great number of serious accidents that have occurred lately—there were four men rendered *hors de combat* at a Rugby match on Monday—may have to do with this. Golf has, on the other hand, become very popular this year.

### STUDENTS' MEDICAL SOCIETIES.

### Middlesex Hospital Medical Society.

A meeting was held on the evening of Thursday, December 11th, Mr. Gordon Brodie, F.R.C.S., occupying the chair. Mr. R. J. Gladstone read a short paper, detailing the results obtained at Middlesex, up to the present time, from the inoculation of tuberculous patients with Professor Koch's fluid—results which, at any rate, were far from discouraging. After describing the symptoms observed in three cases which had been especially under his care, Mr. Gladstone spoke of the changes which were produced on the bacilli themselves, illustrating his point by microscopical specimens of sputum mounted both before and

### Students' Medical Societies—(*continued*).

after the injection had been made. Mr. Gordon Brodie followed with a paper on "Syphilis," in which he laid considerable stress on the manifold ways in which the disease may be transmitted, and the methods which should be used to prevent its spread from the syphilitic to the healthy. Vaccination, as a possible means of infection, was carefully dealt with, whilst hereditary syphilis formed an important item for discussion. Mr. Brodie, after referring at some length to the various modes of treatment in vogue at the present day, brought his paper to a close—a paper which, though working on old ground, was full of interest, amusement, and practical instruction. Messrs. Lee, Baldwin, O'Malley, and Cockill took part in the discussion which ensued.

### Royal Veterinary College Medical Association.

The 138th ordinary general meeting of this Association was held in the theatre of the College on Wednesday, December 3rd, 1890, at half-past six p.m., Mr. Spencer, V.P., in the chair. There were present: The President, Professors Axe and Shane, Mr. E. R. Edwards, M.R.C.V.S., 39 student members, 10 visitors, and the assistant secretary. The minutes of the last meeting were read and confirmed. Mr. S. Bennett then read his paper on "Rot in Sheep." He commenced his very able paper by pointing out the great importance of the disease both to the veterinary surgeon and to stockowners, it being a serious loss to the latter in many parts of the country. He next gave an historical account of the disease, quoting the writings of Markham, Cawshey, Bradley, and many others down to the time of Youatt, who published his first book in 1837. The author next mentioned the kinds of pasture likely to communicate this disease, and also that cold, exposure to wet at the lambing season, coupled with bad food, were predisposing causes. The progress, duration, and nature of the disease was next dealt with, and the animals most likely to be affected were mentioned. The life history of the liver fluke was accurately followed out, and the size and character of the eggs mentioned. The symptoms and pathology of the disease were next discussed, the constitutional and local symptoms being very clearly described. The author concluded his most interesting and instructive paper by referring to the treatment both curative and preventative. A discussion followed, and the author replied.

### PASS LISTS.

### University of London.

M.D. EXAMINATION.

MEDICINE.—H. C. Bristowe, St. Thomas's Hospital; W. H. B. Brook, B.S., St. Bartholomew's Hospital; H. J. Campbell, Guy's Hospital; H. E. L. Canney, University College; G. A. Carpenter, Guy's Hospital; P. J. Duncan, Charing Cross Hospital; F. Edge, B.S., B.Sc., Owen's College and Manchester Royal Infirmary; *W. S. Fenwick, B.S., London Hospital and Strasburg and Berlin; E. B. Hastings, University College; J. A. Hayward, St. Bartholomew's Hospital; W. L. Heath, B.S., St. Bartholomew's Hospital; J. S. Hicks, London Hospital; R. Honeyburne, Royal Infirmary, Liverpool, and University College; W. H. Kelson, London Hospital; C. A. Kent, B.S., B.Sc., University College; G. H. Lang, University College and Manchester Royal Infirmary; L. W. D. Mair, St. Bartholomew's Hospital; W. P. May, B.Sc. (Gold Medal), University College; G. H. Melson, Queen's College, Birmingham; E. Moss, B.S., Guy's Hospital; A Parkin, M.S., Guy's Hospital; B. Pierce, St. Bartholomew's; J. E. Platt, Owen's College and Manchester Royal Infirmary; E. B Randall, University College; C. Read, University College; J. L. Roberts, B.A., B.Sc., Guy's Hospital; T. W. Smith, Guy's Hospital; *E. H. Starling, B.S., Guy's Hospital; G. W. Sutherland, B.A Syd., University College; J. Wheatley, B.S., King's College; H. Williams, St. Bartholomew's Hospital; W. A. Wills, Westminster Hospital; F. R. Blaxall, University College. * Obtained the number of marks qualifying for the gold medal.

M.S. EXAMINATION.

R. D. Mothersole, Guy's Hospital; H. B. Robinson, M.D., St. Thomas's Hospital; J. Swain, M.D., Westminster Hospital; A. H. Tubby, Guy's Hospital.

B.S. EXAMINATION.

FIRST DIVISION.—F. C. Abbott, B.Sc., St. Thomas's Hospital; H.W.C. Austen, St. Bartholomew's Hospital; R. C. Bailey, St. Bartholomew's Hospital; J. O. W. Barratt, B.Sc., University College; T. J. Dyall, St. Bartholomew's Hospital; W. H. Evans, M.D., B.Sc., University College; F. W. Hall, Guy's Hospital; E. V. Hugo, St. Bartholomew's Hospital; A. W. W. Lea, Owen's College and Manchester Royal Infirmary; A. E. Norburn, Guy's Hospital; J. S. Richards, Guy's Hospital; L. Williams, University College.

SECOND DIVISION.—J. H. Bryant, Guy's Hospital; Ellen Margaret Farrer, London School of Medicine and Royal Free Hospital; J. Fawcett, Guy's Hospital; F. Fawssett, St. Thomas's Hospital; S. H. Jones, St. Thomas's Hospital; J. S. McGowan, B.Ss., Owen's College and Manchester Royal Infirmary; T. F. Ricketts, B.Sc., Guy's Hospital; G. H. D. Robinson, St. Bartholomew's Hospital; S. H. Snell, University College; T. G. Stevens, Guy's Hospital; H. Tilley, University College; H. W. Webber, Guy's Hospital.

### Pass Lists—(*continued*).

### Cambridge, Dec. 10.

Second Examination for Medical and Surgical Degrees, Michaelmas Term 1890, Part II.

Human Anatomy and Physiology.—Examined and approved: Allen, B.A., Christ's; Barrett, B.A., Cai.; Blandford, B.A., Pemb.; Blumfield, Cai.; Bottomley, Cai.; Carver, B.A., Christ's; Christopherson, B.A., Cai.; Collis, B.A., H. Selw.; Cuff, Joh.; Dobie, B.A., Cai: Dodgson, B.A., Emman.; Eichholz, Emman.; G. H. Field, B.A., Clare; F. B. Glover, B.A., Joh.; Gooding, B.A., Cai.; Goodman, Joh.; Grove, B.A., Sid.: Grunbaum, Cai.; H. B. Hewitt, B.A., Clare; Hofmeyr, Trin. H.; Hunter, H. Cai.; Ivatt, B.A., Christ's; Kempson, B.A., Cai.; Lawrence, B.A., King's; B. H. Lees, B.A., Joh.: F. H. Lewis, Joh., Locket, B.A., Christ's; Major, B.A., Trin.; M'Cardie, B,A., Cai.; Moore, B A., H. Selw.; Murphy, Cai.; Nicolls, M.A., Pet.; R. E. Nix; B.A., Cai; Noble, B.A., Cai.; Norbury, B.A., Trin.; Peatling, B.A., Magd.; A. S. Ransome, B.A., Trin.; Ransome, B.A., Clare; Richards, B.A., Christ's.; Rogers, B.A., Cai.; Russell, B.A., Down.; Sankey, M.A., Joh.; A. Shillitoe, B.A., Trin. H.; Slater, B.A., Cai.; W. M. Smith, B.A., Cai.; Stewart, B.A. Christ's; Still, B.A., Cai.; Taylor, B.A., Christ's; Waithman, M.A., Madg.; J. C. Webb, B.A., Clare; Windsor, non-collegiate; Woodroffe, Cai.; Wright, B.A., non-collegiate.

First Examination for Medical and Surgical Degrees. Michaelmas Term, 1890. Part I.

Chemistry and Physics.—Examined and Approved: Blatchford, Sid.; Bumsted, B.A., Joh.; Charlton, Cai.; Crampton, H. Cav.; C. D. Edwards, Joh.; Fowke, Queen's; Gaine, Emman.; Gaskell, Christ's; Graham, Trin.; Howitt, Cai.; King, H. Selw.; McCurdy, Pemb.; Marshall, Cai.; Martin, Pet.; Maturin, Cai.; Mayor, Joh.; Moore, B.A., Joh.; Muriel, Down; Myers, Cai.; Paterson, Emman.; Prince, Cai.; Rigby, Cai.; Roberts, B.A., Down; Salt, Cai.; Thomas, Cai.; Townsend, Clare; Troup, B.A., Pemb.; Tuckett, Trin.; Turner, Emman.; Tyrrell, Clare; A. B. Ward, H. Selw.; Wedd, Down; Wilkinson, Emman.; Young, Cai.

Part. II.

Elementary Biology.—Examined and Approved: Ambrose, Clare; Ascherson, Pemb; Bumsted, B.A., Joh.; Burnett, Joh.; Carme, Cai.; C. E. Dashwood, Magd.; Draper, Joh.; C. D. Edwards, Joh.; E. L. Evans, Trin.; Giles, B.A., Pet.; F. A. Godson, Joh.; Gruber, Joh.; R. J. E. Hanson, Trin.; Hey, Trin; Lance, King's; Le Cren, Christ's; Martin, Pet.; Mort, Trin.; Muriel, Down; Myers, Cai.; Nicholls, Joh.; Pearce, Trin.; Pentreath, Queens'; Pollitt, Trin.; R. J. Rowland, Corpus; Hon. G. H. Scott, Trin.; Sedgwick, Clare; Sheppard, Christ's; Thomson, Christ's; Thornely, B.A., Clare; Townsend, Clare; Turnbull, Cai.; Turner, Emman.; Tyrrell, Clare; Wedd, Down; Winkfield, Cai.

Special Examination for Ordinary B.A. Degree.

Zoology.—Class I. (in order of merit).—Barton, Joh.; Higgins, non-collegiate. Class II.—None. Class III.—Nesham, Trin.

### Royal College of Surgeons of England.

The following gentlemen, having passed the necessary examinations and having conformed to the bye-laws and regulations, were at the ordinary meeting of the council, on Thursday, the 11th inst., admitted Fellows of the College, viz.: J. B. Coumbe, M.D. Bruss., L.R.C.P. Edinb., St. Mary's Hospital; E. T. Collins, L.R.C.P.Lond., Middlesex Hospital; R. Caldwell, L.R.C.P.Edinb., Westminster Hospital; W. H. Evans, M.D.Lond., University College Hospital; W. L. Blight, M.D. Durham, L.R.C.P.Lond., Guy's Hospital; C. Williams, L.R C.P.Lond., University College Hospital; F. H. Barendt, M.D.Lond., L.R.C.P. Lond., Liverpool Royal Infirmary; F. F. White, L.R.C.P.Lond., St. Mary's Hospital; W. H. C. Staveley, L.R.C.P.Lond., St. Thomas's Hospital; J. J. Clarke, M.B.Lond., St. Mary's Hospital; J. Murray, M.B.Dublin, Dublin and Middlesex Hospital; C. C. Braine, L.R.C.P.Lond., Charing Cross Hospital; G. L. Cheatle, L.S.A.Lond., King's College Hospital; H. S. Collier, L.R.C.P.Lond., St. Mary's Hospital; F. Xavier da Costa, L.R.C.P.Lond., L. M. and S. Bombay, Charing Cross Hospital; R. Devereux Mothersole, L.R.C.P.Lond., Guy's Hospital; J. G. Colby, M.B Oxon., L.R.C.P.Lond., St. Bartholomew's Hospital; J. P. Parkinson, M.D.Lond., L.R.C.P. Lond., University College Hospital; P. T. B. Beale, L.R.C P.Lond., King's College Hospital; J. O. W. Barratt, M.B.Lond., University College Hospital; J. R. Morison, M.D.Edinb., F.R.C.S.Edinb., Durham and Edinburgh Universities; and G. A. Peters, M.B.Toronto, Toronto University. Thirty-nine candidates presented themselves for examination, 25 of whom acquitted themselves satisfactorily, including three candidates who have not yet attained the legal age of 25 years, who will, therefore, not receive the diploma until future meetings of the council. The following gentleman has passed the necessary examinations, and having conformed to the bye-laws and regulations, was, at the same meeting, admitted a member of the college, viz., Mr. A. C. Lewis, L.S.A.Lond., University College Hospital.

### ATHLETICS.

### Football.

Association Rules, December 10th.

*Medical Staff Corps v. 1st Life Guards.*—There was a numerous company present in Regent's Park to witness this match, notwithstanding the fog. Shortly after the time fixed for the start the Medicals kicked off. For the first few minutes the game was very lively, most of the play being done in the visitors' half. Both teams were fairly representative, and up to half-time no score was registered for either side. After crossing

### Athletics—(*continued*).

over, the Guards pressed, and after fifteen minutes play Naimby obtained a goal. Smith scored again for the home team with a long shot from the centre. For the next ten minutes there was even play, after which Naimby scored another goal for the Life Guards. No further score was registered, and thus the Medicals were defeated by three goals to one. Sides—First Life Guards: Large (goal). Downs and Watson (backs). R. C. Hibbert, Campbell, and Bailey (half-backs), Barber and Corporal Smith (right wing). Smith (centre). Mitchell and Naimby (left wing). Medical Staff Corps: Fitzgerald (goal). Rogers and Birch (backs), Thompson, Kingwell, and Burr (half-backs), Candy and Hiatt (right wing), Varley (captain) (centre), Hayhide and Sullivan (left wing).

RUGBY RULES, DECEMBER 10TH.

*Oxford v. Cambridge.*—Owing to the dense fog which prevailed at the Queen's Club, on Wednesday, the 10th, the annual match between the two universities had to be postponed. This would have been the 18th match between these clubs, and it is the first time that the game has had to be put off. Up to within an hour of the time for the match to begin, hopes were entertained that it would be just possible to play, but the fog came on worse, and the touch flags could hardly be seen, even from the barrier round the ground. A large number of people had already collected to see the most fascinating football match of the season. These received back their gate money and had to depart without seeing anything. Both teams having arranged to go on a tour no date could be fixed for their meeting, but it will be early next term. The sides were:—Oxford—*P. C. Cochran (Oriel and Loretto) (back), *C. J. N. Fleming (Queen's) (captain), *P. R. Clauss (Keble and Loretto), W. H. Parkin (Brasenose and Sedbergh) (three-quarter backs), *R. F. C. De Winton (Exeter and Marlborough) and *R. G. T. Coventry (Brasenose and Hereford) (half-backs), *E. G. H. North (Keble and Blackheath Proprietary), *J. H. G. Wilson (Queen's and St. Peter's, York), *L. J. Percival (Trinity and Clifton), *A. R. Kay (Oriel and Fettes), *A. M. Patterson (New College and Loretto), E. Bonham-Carter (New College and Clifton), W. Rice Evans (Jesus and Cowbridge), S. E. Wilson (Trinity and Liverpool College), and P. R. Caddell (Balliol and Haileybury). Cambridge.—§‖G. Macgregor (Jesus and Uppingham) (back), †‡§‖P. H. Morrison (Trinity Hall and Loretto), §‖R. L. Aston (Cains and Tonbridge), and C. A. Hooper (Trinity and Clifton) (three-quarter-backs), ‡§W. Martin-Scott (Jesus and Craigmount) (captain) and ‡§W. Wotherspoon (Clare and Fettes) (half backs), ‡§‖S. M. J. Woods (Jesus, Brighton, and Australia), §T. W. P. Storey (Trinity Hall and Fettes), ‡§F. C. Bree-Frink (Peterhouse and Sherborne), R. Thompson (Pembroke and Cheltenham), C. P. Simpson (Trinity and Wellington), W. I. Rowell (Jesus and Marlborough), H. W. T. Patterson (Trinity Hall and Wellington), H. Staunton (Selwyn and Bromgrove), C. J. Nichol (Queen's and Llandovery). *Blues. †Player took part against Oxford in 1887. ‡Player took part against Oxford in 1888. §Player took part against Oxford in 1889. ‖ Internationals.

DECEMBER 10TH.

*Swinton v. Edinburgh University.*—This match was played in the presence of about three thousand spectators, on the ground of the former. Shortly after the start Hall passed to Valentine, it was transferred to Winterbottom, and he ran to the visitors' line, and a minor was thus credited to Swinton. The latter team had the best of the game; Hallam gained a try, which Valentine converted into a premier point; then Hall crossed the University line between the posts, and Valentine again succeeded in gaining a goal. At half time Swinton had two goals and two minor points and Edinburgh nil. Shortly after changing ends Valentine dropped a neat goal for the home team; soon after a minor point was gained by the University. Just before time was called Pender ran to the centre, where he transferred to Field, who dashed along the left touch line, and he was only stopped by Valentine when he seemed certain of a try. A minor resulted to Edinburgh after a free kick. Swinton was thus victorious by three goals and three minors to two minors. Sides: Swinton.—Coop, back; W. Winterbottom, J. Valentine, and J. Mills, three-quarter backs; F. Green and W. Bumby, half-backs; N. Hotchkiss, A. Paul, T. Hallam, F. Lomax, T. Walker, S. Hall, W. Evans, A. Sharples, and J. Hotchkiss, forwards. Edinburgh University.—T. Dunlop (Irish International), back; G. Bearne (Somerset), J. J. Mitchell (Cumberland), and A. Field (Bradford), three-quarter-backs; J. Pender, captain (Edinburgh Inter-city) and G. S. Thom (Scotch International), half-backs; H. Theodore (Owens College), W. J. Davies (Irish International), W. F. Menzies (Scotch International), Johnson (United Hospitals), H. T. Thompson (Old Leysians), H. Cameron (Fettes School), J. Legatt (Edinburgh City), Roderick (Edinburgh City), and Gunter (Dover). Referee—Mr. E. Siddon (Broughton).

### Golf.

*Cambridge University v. Royal Epping Forest.*—The competition between these clubs was decided on Saturday at Cambridge. There were 18 aside. One round of 18 holes was played. The day was fine, though dull, and the putting greens were in good order. The University won by 37 holes. Score: Cambridge University, 60 holes; Royal Epping Forest, 23 holes.

### EVENTS FOR THE MONTH OF DECEMBER.

[Items intended for this column must reach the office, 140, Strand, W.C., not later than first post on Monday mornings.]

**December Meetings and Athletics.**

For list of Students' Medical Societies' Meetings and Football Matches during December, see THE HOSPITAL for Nov. 29th.

### GENERAL CHARITIES.

[This Column is devoted to an account of work of charitable institutions other than Medical Charities. The Editor will be glad to receive reports or news of work at 140, Strand. *Philanthropy* should be plainly written on the envelope.]

The Court of Common Council have made a grant of 100 guineas in aid of **THE ASSOCIATION FOR THE ORAL INSTRUCTION OF THE DEAF AND DUMB**, 11, Fitzroy Square, W.

There probably was no time when the work of **THE CLERGY ORPHAN SCHOOLS** was more needed than it has been during the past few years. Since the foundation of the schools in 1749, over 2,850 children have been admitted to their benefits, and the schools, which were built for 220 now, contain 200 pupils who are elected by votes. On leaving the schools both boys and girls are assisted to further education or to make a start in life by means of an apprenticing fund. Secretary, Rev. H. Wesley Dennis, 62, Lincoln's Inn Fields, W.C.

A special fund for work among the young is now being raised by **THE CHURCH OF ENGLAND TEMPERANCE SOCIETY**, and donations amounting to nearly £800 have already been promised for this purpose. This widening of the scope of the society's work naturally entails increased expenditure. In order to carry on the work efficiently a staff of trained lecturers is required. It is hoped that the preventive work which the Society intends to pursue will contribute largely to the safeguarding of the young against temptations which will beset them later in life, and the evil results of which to the nation at large are only too visible. Donations to this fund should be sent to the Secretary, Junior Division, C.E.T.S., 9, Bridge Street, Westminster.

The system which makes the admission of candidates for the benefits of a charity depend upon the number of votes given to the applicant is fundamentally wrong. It is pernicious in many ways. The most deserving applicants are only too often last in the race; they are kept waiting out in the cold because they have not sufficient influential friends, and they are frequently put to an expense which should not be imposed upon such people. The system is almost a degradation of the word charity, for where there is a consideration in return for money given there is no charity in the true sense of the word; the so-called donation is no gift, it is the purchase of a privilege, and of a form of patronage which does little honour to the holder. It is a regrettable fact that many people want a more tangible return for their money than the mere knowledge that the gift is likely to be of benefit to those who have failed in the struggle for life, and that so many otherwise excellent institutions are carried on on these principles. A commendable feature in the administration of **THE ASYLUM FOR FATHERLESS CHILDREN** at Reedham, near Purley, in Surrey, is the fundamental rule, beyond the control of any meeting or any future act of incorporation, that while the education of an infant shall be strictly religious and Scriptural, no denominational catechism whatever shall be introduced, and that no particular forms shall be imposed on any child contrary to the religious convictions of the surviving parent or guardian of the child. Infants of three months old are eligible for admission, and children are received up to the age of eleven, and are retained until they are fifteen years of age; but no workhouse case is eligible for the charity. In special cases children are deemed eligible whose father is entirely and permanently disqualified, mentally or physically, for the duties of life, provided in all cases that diseased, deformed, or infirm children are inadmissible. There are over three hundred children in the asylum. On leaving many have entered business houses in the city, the majority, in fact, appearing to become office clerks, and to enter shops. The subjects taught in the boys' school include reading, writing, spelling, geography, and map-drawing, English grammar and composition, history, shorthand, French, elementary German, mathematics, experimental science, vocal and instrumental music, gymnastics, and swimming. The girls, who number nearly one hundred, are taught, in addition to the ordinary elements of education, English history and literature, domestic economy, botany, vocal music, freehand, model, scale, and geometrical drawing, French, calisthenics, needlework, and cookery. Why English literature is considered a necessary study for the girls and not for the boys we are not aware. The majority of girs on leaving become teachers, many become milliners or enter shops, and some enter the Civil Service as Post Office and telegraph clerks. The asylum is meant for children of the poorer classes. Secretary, Mr. J. Rowland Edwards, 35, Finsbury Circus, E.C.

